# Ritual Slaughter: The Tradition of Pilot Whale Hunting on the Faroe Islands

**DOI:** 10.3389/fvets.2021.552465

**Published:** 2021-04-09

**Authors:** Hanna Maria Mamzer

**Affiliations:** Adam Mickiewicz University, Poznań, Poland

**Keywords:** whaling, hunting, pilot whales, tradition, ritual slaughter, halal/kosher slaughter, diversity of cultural traditions

## Abstract

Faroese people consider grindadráp, the hunting of pilot whales, as a part of their cultural heritage, but from the point of view of veterinary sciences and biology, the method of killing pilot whales is a form of a ritual slaughter performed on fully conscious animals that are aware of their circumstances. Pilot whales are social, intelligent, and communicative animals that demonstrate complex social behaviors. Therefore, this traditional whaling method should be considered as a procedure in which animals are exposed to high levels of distress. In the context of contemporary civilizational development and material welfare, the practice of whaling may appear to be an inadequate and cruel relic of the past. This text explores social and cultural issues caused by pilot whale hunts and presents an understanding of the term tradition and some perspectives of how traditions change. The specificity of pilot whales as a species is presented, setting a foundation for a discussion about hunting itself. The conclusion of the text discusses different social perceptions of grindadráp by presenting arguments for and against the hunting. This analysis includes a presentation of actions undertaken bywhale hunting opponents.

## Diversity of Cultural Traditions

The issue of cultural diversity is already well-embedded in the social sciences and humanities, but the changing reality implies new areas of research. One of these concerns culturally differentiates attitudes of people toward other animal species. This differentiation requires further reflection. Changes in the functioning of postmodern human societies require that the social perception of tradition and attitude to cultural practices involving the inclusion of non-human animals in human activities be modified as well. Sociological concepts generalizing trends in social change indicate that postmodern communities put very little emphasis on tradition and that tradition ceases to be an important element that binds communities together. Moreover, the integrating function of religion, language, and the idea of forming small social groups based on blood ties diminishes. Local communities are also constructed on the basis of a conscious selection of members rather than on the basis of a cultural message that is the subject of thoughtless cultural transmission from one generation to another. Neo-traditions, or rather some kind of individualized habits, preferences of the participants modified on the basis of individual choices, (1) fulfill the function of group traditions and are increasingly becoming common practice. These individually created customs become the base for a sense of ontological security, and thus a kind of belief in the predictability of the world, a belief which is necessary for a person to function effectively every day. This leads to the popularization of trends where people make many more choices (than in traditional societies) about how they will function, how they will live, and what their values will be (2). This is particularly true for young people in adolescence and shortly afterwards, but older members of postmodern societies function in a similar way: they have to, because it is imposed by the variability of everyday life.

However, it is equally true that in the face of intense globalization changes, there are trends aiming at re-cultivating old traditions or selected customs, very often modified in such a way as to constitute an adequate reference to social reality (3). Their sociocultural function is the same as that of the traditions cultivated in traditional societies: they are meant to integrate, create a sense of community, generate a sense of predictability of the world, and provide a type of reference point for the values to be respected. A part of the diverse traditions still cultivated takes on a purely symbolic form and has a very modest or even no pragmatic meaning. An example of this kind of tradition in the Roman Catholic Church is posting information about the people who are planning to get married on the church notice boards, with the assumption that outsiders who are aware of possible contraindications to such a marriage will report them to the priest. Such a tradition was reasonable and fulfilled its socially controlling function in small, homogenous, steady, and unchanging local communities where all or almost all participated in ecclesial practices and knew each other. Such a tradition does not fulfill its function in heterogeneous postmodern, atomized, and anonymized communities with a significant degree of anomie, because the communities are too large, their members do not know each other and, above all, they do not all participate in the same kinds of religious practices. Such a function of posting marital announcements could still be implemented relatively effectively using a suitable medium (the Internet), but its traditional character and method of implementation do not match the reality of the functioning of the contemporary postmodern society. It is therefore an example of a kind of ritual action that has a marginal practical significance.

Some selected traditions and customs are, however, taken up and cultivated by people as an expression of their own identification with a particular community and as an external manifestation of their own identity embedded in a particular community. Wearing a hijab by Muslim women living in Western European countries (4) is a method of demonstrating one's individuality within the group, based on autonomous choices. Social behaviors voluntarily undertaken by people in different cultures, such as the owning of a Faroese passport (5) or participation in grindadráp (also known as grind, the hunting of pilot whales in the region of the Faroe Islands), are similar examples (5, 6). According to Fielding enjoyment of grindadráp as well as a higher consumption of blubber are interrelated with the holding of a Faroese passport, which may be interpreted as a stronger connection to the traditions in Faroe Islands.

I propose an interpretation of the term tradition as a process of intergenerational transmission of some kind of cultural content (among others: beliefs, judgments, customs, mindsets, and behaviors), recognized by a given human community as socially important for its present and the future. The social and cultural functions of the cultivated traditions remain unchanged: the integration of the community by indicating the shared similarities and by distinguishing it from external communities; the control of its members (the individual communicates his or her affiliation to a group by participation or a denial of participation, but the exclusion of the individual from participation in the tradition is in turn a message to the community about the status that the individual has in the chosen range); the possibility of identifying “insiders” and “outsiders” (ignorance of the ritual is an unambiguous message that the individual is an “outsider”); and the implementation of rituals of transition, etc. The function of tradition is undeniably important for humans, from the point of view of both social and psychological functioning (1, 7).

However, in the context of the functioning of postmodern culturally diverse societies, the need to revise the forms in which tradition is implemented becomes particularly important. It should be pointed out that cultural transmission causes a modification of traditions; these traditions almost never retain their original forms. Moreover, traditions change so much that it is often difficult to discover the original meaning of a certain type of behavior. They are often shifted from the sphere of performative activity to the linguistic sphere. They then do not function as a behavior but as a verbal practice (such relics of the past are hidden in proverbs, adages, and parables, which on the basis of culturally embedded practice are often undertaken without reflection; their meaning is deeply hidden). In relation to pilot whale hunting, Bulbeck and Bowdler (6) indicate changes in the way hunting is conducted [e.g., the use of fast motorboats in “traditional hunting” (8, 9)]. During a period of intense globalization, when the unification of cultures is at an advanced stage, the preservation of traditions has an important dimension for the maintenance of the social identity of the community. However, with the civilisational changes taking place, numerous practices are perceived as inadequate in relation to the overall character and dimensions of global human culture. The modification or even abolition of such practices is controversial. It is particularly visible in the context of issues related to the possibility of preserving the group identity of cultures that have become victims of the oppressive actions of dominant communities colonizing on the basis of cultural imperialism. Here the discussion on cultural practices takes on particular importance because traditions become the foundations of autonomy and the bastions of resistance. Changes in traditional practices result from a process of reflection on their meaning and adequacy: the initial stage is to raise the issue, identify it and then to discuss it and finally modify it, through various social practices, including legal regulations (10).

Drawing borders, indicating where the demarcation line between which cultural practices are acceptable and which are not, is extremely controversial and culture dependent. For instance, the nation of Buryats in Mongolia observes a rule of humanitarian, in their perception, slaughter of horses sacrificed during the annual holiday of “tailgan.” The tissues of the abdomen of the horse are cut and the beating heart is ripped out from the still living animal (11). Controversies related to the level of acceptability are also caused by the Chinese custom of consuming wild species of animals (12). It should be assumed that most probably there will always be a group of people who will find the proposed division between what is acceptable and what is not unfair. However, this cannot justify the abandonment of the subject. In such circumstances, at least an attempt should be made to define what is and is not accepted in the context of cultural differences. Establishing one single rule is an enormous challenge. However, I propose setting a limit based on whether the practices in question involve physical damage to the shells of a human body or another animal against their will. This is a radical criterion, but its adoption would make it possible to objectify the tool for assessing cultural practices. In the proposed criterion, the violation of body shells could be treated as acceptable, but it would have to take place with the conscious consent of the interested party. The simplest scenario seems to refer to a conscious adult who, knowing what may happen, agrees or disagrees. However, how to assess certain cultural practices that are physically painful, but are psychologically and socially satisfying (e.g., certain rituals of transition, such as scarification) is more challenging. This should not be a controversial issue as long as the subject of the action agrees to it. It is obvious that practices interfering with physical integrity could not be taken against children or non-human animals (the question remains, of course, how to regulate, e.g., the intervening medical treatment). This is a critical question that requires an in-depth reflection.

Some cultural practices trigger more robust protests and significantly more negative evaluations (e.g., girls' cliteroctomy) than others (e.g., ear piercing); in the case of non-human animals: disbudding is evaluated much more negatively than, for example, ear tagging. The problem is to induce psychological interactions which are perceived as negative or oppressive by the subject on which they are executed.

It is therefore difficult to attempt to establish an objective criterion indicating which activities are acceptable and which are not in relation to other living beings, but this seems to be a suitable path for an egalitarian approach to cultural differences in the treatment of non-human animals.

Currently, it is impossible to base identity on features attributed by the community in the context of treating identities, including social and cultural identities, as processes rather than states, based on autonomous, more or less conscious choices. Identities are still important, but in a flowing postmodernity, elections of identity-critical elements are ongoing. As the classics of contemporary considerations of identity claim (1, 7, 13–15), identity itself is therefore also a variable and a task to be accomplished. It is not received from any community: it is a subjectively built structure consisting of elements obtained through socialization, its own activity, and a kind of genetic heritage. Embedding identity in socially constructed practices (including traditions) is ineffective because the practices undergo changes—and more importantly, these changes occur very quickly (which was not the case in traditional communities).

In reflections on identity issues, it is indicated that the consciousness of a tradition's continuity is important for the preservation of a tradition: tradition remains a tradition as long as its historical variability is documented in some way and embedded in social awareness. An ideal example is the parable of the mythical ship of Theseus. The ship underwent consecutive repairs and finally did not contain any original parts, but still “was the ship of Theseus.” Traditions can be said to be identical: they change under the influence of social processes and can ultimately take forms that are radically different from their original versions. This is an indication of the possibility of modifying a tradition, including its intentional modification. Invariability does not make something a tradition. The tradition is determined by the continuity of the process.

The above context is drawn in order to set a background for the consideration of cultural practices and their acceptance in terms of social and individual aspects, proposing a division into acceptable and unacceptable, on the basis of whether the entity on which the practice is implemented can maintain its integrity, in particular its physical integrity. The examples that are particularly prominent in this context are such cultural practices as *corrida de toros* (16) or Faroese pilot whale hunting, the subject of further discussion in this text.

## The Pilot Whale as a Subject of Human Cultural Practices

Pilot whales (*Genus Globicephala*) are mammals of the dolphin family (*Delphinidae*). Pilot whales are two separate but very similar species: long-finned and short-finned. Their name derives from the typical round shape of their heads. The generic name is a combination of the Latin word globus, “ball, sphere,” and the Greek word κ*εϕαλη* kephalē—“head.” These animals live in all oceans and seas, at temperatures between 0 and 25°C, and their prevalence has certainly contributed to their hunting, although the exact population size and growth rate are unknown. The total number of pilot whales is estimated to be around 780,000 individuals living in the North Atlantic, but the inaccuracy of the data means that in the IUCN (International Union for Conservation of Nature) reports that long-finned whales have the status of “missing data” (17). Moreover, studies show that in some regions the populations of long-finned pilot whales are very small. In the Gibraltar region, the developmental assessment of the current 250-member population showed an 85% probability of extinction of the population over the next 100 years, due to viral diseases that decimated the population in 2006 and 2007 and factors resulting from anthropopressure: climate change, increasing maritime traffic, increasing environmental pollution, and the impact of the fishing industry (18, 19).

Long-finned pilot whales (*Globicephala melas*) are an object of particular desire on the Faroe Islands. Traill (20) described this species, one of the largest members of the *delphinidae* family: males reach a length of up to 8.5 m and a weight of up to 3.5 tons and females up to 6 m and a weight of up to 2.5 tons, respectively. The coloring of the animals ranges from metallic gray to black, with light gray or white marks on the throat (in the shape of an anchor) and abdomen, in the form of a smudge behind the eye, and around the dorsal fin to form a so-called “saddle patch.” The thick dorsal fin is sickle-shaped and lies within one third of the length of the animal's back. The name of the long-finned pilot whales refers to their long, sickle-shaped pectoral fins, whose length reaches 18–27% of the whole body length (21). Pilot whales have one blowhole.

Long-finned pilot whales maintain very strong social ties and are social animals. They form groups of several dozens to a thousand individuals (22–24), although the most common groups are composed of several dozens to a 100 individuals. Pilot whales feed on small fish and squids. Females mature at the age of 8 years, males at the age of 12 years. Pregnancy lasts up to 16 months and the female gives birth to up to five calves during her life. Reproduction occurs rarely within one family group. Most often fertilization occurs when alien groups meet. This can happen all year round, but most often in spring and early summer. Calves are born mmeasuring around 2 m long and weighting about 75 kg.

Social groups formed by long-finned pilot whales are basically very complex families, in which the matrilinear system functions. The families are composed of adult females and their offspring (25). Mature males remain in their maternal families (26), but do not reproduce there and often change families, joining other than their own families of origin. Research on short-finned pilot whales (*Globicephala macrorhynchus*) and killer whales (*Orcinus orca*) has shown that their females live long after the end of the period of reproductive capacity, which is an exception in the animal world (a similar pattern applies only to *Homo sapien*s). Females in menopause take the lead in the herd, especially during the salmon hunting season, and especially when the salmon population is small and hunting is difficult. Above all, their role is to pass on knowledge about the ecosystem to the pod and the threats resulting from the specific nature of the environment: the transfer of knowledge on this subject may contribute to the blooming of the population and to the success of reproduction. This tactic justifies the long life of the female killer whales and short-finned pilot whales. Whether this kind of behavior is also present in long-finned pilot whales is still unresolved (27). The fact that they repeatedly become victims of hunting in the same places may indicate that the females of long-finned pilot whales do not provide the pod members with information about threats (or are not able to identify these threats). The process of transmitting such knowledge is certainly impeded by the fact that the Faroese try to kill the entire pods, so there are rarely survivors who would be able to pass on the knowledge to other animals.

Long-finned pilot whales are very active and energetic. In young individuals, jumping out of the water is a common behavior, but it disappears with age. In search of small fish or squid to feed on, they are able to stay underwater for almost 10 min and dive to the depths of 600 m below sea level, but do not usually dive deeper than 60 m.

The basis for effective social functioning is communication, which is very well-developed in pilot whales. They differentiate between sounds emitted by other marine mammals that are predators, but, as shown by studies conducted on the behavior of short-finned pilot whales, these animals swim to the source of sound emission, increasing the risk of falling prey to the attacker (28). The intensity of vocal communication is higher during and shortly before feeding but decreases significantly during movement and rest. The intensity of communication also decreases when animals can be close together, which obviously reduces the need for acoustic contact (29). Research also shows that the complexity of vocal communication of long-finned pilot whales locates them among animals with the most complex vocal repertoire. This repetoire consists of numerous whistles, buzzes, clicks, and pulsating and non-harmonious sounds produced singularly or in complex layouts consisting of several segments or elements (30). The abovementioned researchers identified 129 different types of sounds among which they distinguished 29 subtypes and described new, previously unrecognized types of ultrasound used in communication. Pilot whales create their own communication dialects, characteristic of particular social groups (29, 31). The level of complexity of this communication locates pilot whales in the forefront of mammals—but at the same time there is much to be studied yet. In situations of exposure to disturbing sounds, depending on the type of noise, the animals in question used different behavioral strategies but always increased the pod proximity between individuals and gathered in larger groups. When the sounds usually associated with tagging animals with gps transmitters were emitted, the level of pod synchronization increased as well as their closeness and the animals were quieter and restricted communication (21% of cases). The emission of recorded sounds produced by predators, such as killer whales, caused a stronger call and gathering of pilot whales into smaller groups, which swam together to the source of the sound. This type of behavior should be interpreted as a response to disturbing situations resulting in the demonstration of adaptive behavior aimed at defense against predators. All the behavioral tactics observed, despite their different alignment to the stimuli, were designed to reduce the risk of the group losing coordination (32). It seems that this fact of pod clustering is used during pilot whale hunting: frightening (with motorboats or noise) facilitates the killing of animals that are close to each other.

Approaching each other, shortening the distance, and coordinated swimming have a double function for pilot whales: firstly, protection from the threat, but secondly, these actions also fulfill the needs of affiliation and allow the animals to feel the close presence of other individuals, which plays an important role in strengthening the social bonds (33). The high social competencies of pilot whales are also documented in films illustrating individual stories of animals that were captured by humans. An example of this kind is the story of a pilot whale named Sully, who due to hearing impairment was taken care of by a human in Curacao in July 2009. The documentary film about this animal presents the great social skills of building relationships with another species (human), a high level of effective learning based on instrumental conditioning, and strong sensory sensitivity, including touch (positive reactions to stroking, reminiscent of the behavior of a dog lying on its back).

Undeniably, long-finned pilot whales, like other species of dolphins, are very highly developed animals and human knowledge about their lives and the way their senses function is very limited. Our inability to feel what it means to be in their skin limits our capacity to understand these fascinating and mysterious animals. This problem was outlined in 1974 by Thomas Nagel in his essay “What Is It Like to Be a Bat?,” in which he pointed out the inability of humans to experience the world as other living species do. Based on the available knowledge about whales, one should consider them very carefully as “something,” because there is a high probability that they are “someone.”

## Pilot Whale Hunting: Historical Outline and *Grindadráp* Today

The Faroese term *grindadráp* literally means pilot whale slaughter, as the word “*dráp*” means “slaughter” (34). The history of whaling in the area of the Faroe Islands goes back to the sixteenth century and is deeply connected with community life in the Islands. Most likely, the tradition is older than that. Pilot whales were hunted not only in the Faroe Islands, but also in Japan, Norway, Greenland, Ireland, Newfoundland, the Caribbean, and Cape Cod. Currently long-finned pilot whales are hunted only in Greenland and the Faroe Islands (35). There are 23 specific designated beaches in the Faroe Islands where grindadráp can take place (9).

The Faroese hunt on average 900–1,000 pilot whales yearly (36) and the International Whaling Commission claims that the contemporary level of kills is estimated as about 0.1% of the population, which can be most likely considered as sustainable by the International Union for Conservation of Nature (IUCN) (35). At present the population figure of 778,000 is accepted by the International Whaling Commission's Scientific Committee. Supporters of the hunting tradition claim that this is an estimated number and most likely the population is much bigger. If those estimations are true, even killing entire pods of pilot whales would not seem to threaten the population. Pilot whales are not a species at risk of extinction. They are considered as a least-concern species according to the International Union for Conservation of Nature. They do not qualify as threatened, near threatened, or conservation dependent. The accessibility of animals for the hunts does not seem to decrease, despite the fact that there are entire pods of them killed during hunts.

The whaling records presented by Fielding [(34), p. 289–93] demonstrate changes over the years (the statistics cover the period between 1587 and 2017). Accurate data are available from National Whaling Statistics, Føroya Náttúrugripasafn. This data can also be compared with data provided by Sea Shepherd, a strong opponent of the grindadráp.

The largest documented pilot whale hunting ever recorded occurred in 1940 when 1,200 animals were killed [(37), p. 54]. The most intensive hunting takes place in the summer between June and October when the whales are most numerous near the shores (24). Groups of up to 200 individuals are then killed (8, 9).

Pilot whale hunting is basically a typical “battue” (10, 34). When a pod comes to the bay and is spotted, one is obliged to inform the community about it. The Faroese interrupt all their activities and run to motorboats, which are then used to lead the animals to the beach where the whales will be killed. Participants do their best to push all the present animals ashore, with no nets, just using noise, boats, and manpower. Animals are killed after being pulled to the beach, usually all of them. As highly social, they are unable to manage on their own. Animals left alive do not swim away for a long time. They swim in a bay full of blood, trying to accompany the killed family members (38, 39). After a hunt in Vestmann on August 27, 2019, the four remaining pilot whales were swimming in the water of the bay for at least an hour and a half after the other members of the pod were killed. The entire hunt lasted 5 h; the actual process of killing 98 animals-−12 min. First, the leader of the pod is killed (the female leader, given the matrilinear leadership scheme among pilot whales). The communication of the rest of the animals is made more difficult by humans who generate noise by banging wooden sticks on the water and hitting the sides of the boat.

Once the animals are forced to the beach, people enter the water and attach to the whales' blowholes metal hooks tipped with a metal ball on one side and ropes attached to them on the other side. The hooks are used to pull the animal into the sand (which must be painful for the animals). There, once relatively stable, the animal is killed by inserting a type of double-sided blade into the spinal canal of the spine (according to the guidelines, the blade should be inserted between the skull and the spinal atlas to break the spinal cord and then shifted to the right and left to cut the blood vessels that supply blood to the brain of the animal). The blade is 4.7 cm wide and the spinal canal is about 5 cm in diameter. The animal does not lose consciousness and does not die as a result of cutting the spinal cord, but as a result of the gradual exsanguination. The neck and sides of the neck of the animal are also cut with a long knife in order to get rid of the blood (with the purpose of improving the consumption properties of the meat). Dead animals are often dragged in the water to the harbor where they are trimmed. After pulling the whales out of the water, the animals are arranged in rows, their stomachs are cut to cool the carcasses, then the length of the animals is measured, which allows for their classification in the appropriate size category (the appropriate number is cut on the side fin). For scientific research, the front part of the mandible that has been cut off (in order to determine the age) and the liver are collected. A consecutive number is cut on the animal's cheek to allow the counting of the animals. Pregnant females are often set aside and trimmed later, when fewer people from outside the community witness this process, as the killing of pregnant mothers raises huge controversies and protests from the opponents of hunting. Pilot whale meat and fat is shared by hunters among all the members of the community who have taken part in the hunt. Raw meat is generally not sold, but dried pilot whale meat can be found occasionally in the markets of Miklagard and in fish kiosks (for example, in Klaksvik). The price depends on the size of the dried piece, but usually the range is 50–100 Dkk (about 15 USD). The idea of distributing meat for free to members of the community (5) is an important argument used to justify the meaning of this type of hunting.

Not all of the carcass of the animal is used. Except for meat from the carcasses and fat, the other parts of the animal (the fins, the head) are not used. They are thrown directly into the sea, where they are to be naturally biodegraded. After the grindadráp on August 27, 2019, Sea Shepherd activists managed for the first time to document the location of the discharge of animal residues directly into the fjord. Such procedures are clearly against the rules, which require that the remains after a whale drive be removed within 24 h after killing and that the quay be cleaned again (40). These residues could be used as feed in the fishing industry (e.g., salmon aquaculture), but unofficial comments from the fishing industry indicate that salmon producers do not want to use this raw material to avoid controversies that could have a negative impact on the sale of their products.

Pilot whales' strong social ties prevent other members of the pod from leaving the wounded or killed animals. They follow the pod and thus often also die (38, 39). This strong social bond was previously utilized by the hunters. Historically, the beaching was accomplished by a boat-based whaler using a spear to stab one of the whales in the tail, causing it to rush forward, toward the beach, in the hopes that the rest of the pod would follow. This strategy exploited the social nature of pilot whales, which are known to remain together in pods even when some individuals are in danger” [(39), p. 6]. Around 400-1,000 pilot whales are slaughtered every year in this manner. Currently, these hunts are not economically justified, they are only an element of the tradition. The Faroese supporters of this tradition defend it as their own cultural heritage, but for many reasons it seems that this tradition should be reviewed (8, 9).

## Damage to Animals

The biggest, objective and undisputed loss for pilot whales during the grindadráp is the loss of life. However, this process is additionally accompanied by the somatic and psychological suffering of animals, which is rarely mentioned in the general debate on this subject. Existing regulations defining how to legally kill pilot whales psychologically can be treated as a rationalization, a process aimed at suppressing the emotions that accompany the killing. A precise description of procedures and tools creates the illusion of control over reality. It does not take into account the sensitivity of animals, nor does it reduce their “bad state” [Gzyra (41) coined the term “badfare” as opposed to “welfare,” as he claims that in the cases of some human practices toward other species, one cannot speak of any level of welfare; according to him, using the term welfare is a hypocritical type of euphemism]. Writing down procedures creates an impression only that the process is under control and that it takes into account the welfare of the animal (10, 42). The quantification of the process—applying measuring strategies, monitoring numbers—are ways of presenting the hunt as a rational, controllable process. This is partially true. But partially it has to be admitted that the hunt, in the water, where a number of people and a number of whales are involved, is a very dynamic phenomenon. Keeping strict control over the entire process all the time seems to be a very unlikely prospect. The idea of controlling the process contradicts the emotional aspect of the grindadráp: it is emotionally engaging for both parties, whales and humans. Therefore, an understanding of the phenomenon requires admitting that it is impossible to control the grindadráp in all aspects, all the time. In addition, observing the killing of the animals is a source of distress for those not involved in the hunt. The argument that the killing is controlled through guidelines is often used by supporters of these hunts.

The information provided above indicates that human knowledge about the various dolphin species is still very limited and that human perception of their specificity and ability to function is mediated by human cognitive abilities. Research indicates that these animals belong to the categories of social species, that they have highly developed and complex communication skills and systems, and that they are intelligent and understand the changes that occur around them. They are also able to communicate about environmental changes to other members of groups. In fact, it should be admitted that it is not known exactly who we are slaughtering (and the notion “who” is used here deliberately, taking into account the complexity of whales' psychological, social, and cognitive functioning). The abilities of pilot whales are still a mystery to the human being. The functioning of all their senses and their brain processes are incomprehensible and we do not know how much these animals understand and how they perceive the world. A translation of their perception of their surroundings into the human categories of perception is still an unfinished task (43). Undoubtedly, hunting constitutes a source of great stress for these whales (44–46). As animals perfectly versed in the environment with an understanding of their surroundings and sensitivity to the social context, they experience an extreme level of emotional arousal during the chase and the killing. This is reflected in studies on the level of cortisol in the body and behavioral and veterinary observations of cetaceans (38, 42, 47, 48).^1^

The North Atlantic Marine Mammal Commission “Instruction Manual on Pilot Whaling” from 2014 clearly indicates that the insertion of the transport hook into the blowhole of pilot whales should be restricted and not abused (50). This is a euphemistic term that conceals the fact that placing this hook and using it to pull an animal weighing up to several tons to the shore must be physically painful for the animal, especially considering the innervation of the body and the very high sensitivity of these animals to touch.

Hunters do not always succeed in stabbing a knife blade into the spine the first time, and multiple stabbings are not uncommon, especially if the animals are moving. Immobilizing the animals is not an easy task. Photographs from the grindadráp point to wounded animals who, while fighting for their lives, hit the boats or rocks and are injuring themselves.

Controversy arises also from the slaughter of numerous animals in a very short time (during the hunt in Vestmann in August 2019, 98 animals were killed in 12 min)—it is obvious that in such conditions there is no possibility to control the process and that it is unknown when the animals die and whether they are dead during further procedures because nobody examines their blinking responses (which is recommended by the manual) (51).

All animals that are being killed know that other members of the pod are also being slaughtered: animals communicate with each other both vocally and behaviorally. It is obvious that they understand what is happening (42, 47). Such practices, which expose animals to stress, are considered unacceptable, both in slaughterhouses and in laboratories. Legislation regulating the killing of animals emphasizes the need to reduce stress and the feeling of pain. Moreover, in both slaughterhouses and laboratories, animals to be killed must be isolated from other animals, in order to not witness the actual process of killing.

Another source of suffering for pilot whales killed during the grindadráp is the psychological harm, which is very difficult to demonstrate objectively, but the occurrence of which is obvious when the specificity of the socio-psychological functioning of these animals is taken into account (52). The prolonged chase that physically exhausts them is also a strong stressor resulting from the desire to preserve life and avoid danger. The biological survival drive is the strongest drive of all. Stress connected with the threat to life is intensified by the fact of being an understanding and conscious witness to the process of the killing of an entire family group. Animals that somehow manage to survive the hunt, as said before, do not leave the pod. They remain in the water blending with the blood, confused, disoriented, looking for their companions, eventually leaving to open sea. This is a particularly distressing experience for a sensitive observer. I have not managed to find any publications describing any monitoring procedures that are enforced to check what happens with survivors; therefore, it is not known if these animals survive and join other groups of pilot whales or if they are likely to die as a consequence of the stress they have experienced.

Animal slaughter is criticized by animal rights defenders, but ritual slaughter, in which animals are killed without stunning, is treated by them as an extreme cruelty. Unfortunately, killing pilot whales during grindadráp is a process of this type. To outside observers, a “calming” impression can be made when an animal pierced with a dagger stops moving, because it seems to be dead. This moment creates the impression of an instantaneous death. It is known, however, that pilot whales die as a result of gradual exsanguination. The incision in the animal's body in order to cut the blood vessels takes place when the animal is still alive. It is undoubtedly a source of somatic and psychological suffering, as a result of direct interference in the integrity of body shells (52).

The method of killing pilot whales described in the binding manual gives the impression that the process is humane. It is not. It cannot be, and all euphemisms used (such as “using a hook should be reduced to a minimum”) serve only to disguise the entire process. While the physical suffering of animals seems to be easier to describe and to measure with the level of secretion of stress hormones, a psychological description of the state of animals chased for hours and then killed is still an impossible task (52). The question of what constitutes the humane killing of animals is a broad theoretical issue which of course requires deeper debate as well. Despite cultural differences in understanding what is a humane death, the term itself implies that the death is understood as an “acceptable way of killing” in human categories. Death is an experience that presents a number of challenges in researching its psychological aspects; therefore, it is almost impossible to define the feelings accompanying it. Undoubtedly, however, it is clear that animals do not want to die, since they mobilize their potential to survive threats to their lives.

## Social Perception of Hunting for Pilot Whales on the Faroe Islands

For humans as creatures for whom vision is the strongest sense, the visual aspect of the grindadráp is undoubtly the element triggering most protest. The strong color of red blood mixed into the waters of the bays, the roiling water during the actual killing of the animals, the number of motorboats, people, and pilot whales involved in the grindadráp, the dynamics of the actual killing of the animals, and, last but not least, the large carcasses of animals lying in the harbor contrasting with the silence and stillness of the picture—all of these cause disturbing reactions in humans. In addition, humans tend to feel a stronger emotional bond to certain types of animals, among them, pilot whales (53).

The perception of hunting depends on the characteristics of the social community assessing the hunt. Different categories of people represent the following types of views:

1. Acceptance of hunting as an element of local culture.
2. Aversion to pilot hunting, as dictated by the reasons indicated earlier: the inappropriateness of this tradition for the civilisational and social development of the region; the negative effects of these hunts on animals; and awareness of the controversial nature of hunting.
3. Definitively negative, presented by pro animal activists, especially Sea Shepherd or Greenpeace.
4. Scientifically justified rational discussion on the legitimacy of killing pilot whales and consuming their meat.
5. Whitewashing of the process, presenting it in a positive manner (emphasizing the elements referring to the specificity of people's activities during the regatta, and not those characteristic of hunting; this is particularly visible in the strategy of reports in the local media.
6. Defensive and offensive publishing activities presented by the media, aimed at active defense through attack. For example such as “Is civilization being controlled by Eco-terrorism?,” by Hansa J. Hermansen (Local issue no 3/2019).

The narrative supporting the continuation of pilot whale hunting is based on the following arguments:

1. Hunting provides food that is sourced in a manner that minimizes environmental impact and preserves the principles of sustainable development (54). The Faroe Islands do not have agricultural land, because the terrain and soil structure make it virtually impossible to cultivate crops (apart from small domestic crops). Vegetables, cereals, and, in principle, all major food products are imported. The Faroe Islands have extensive fishing and aquaculture salmon production and sheep farming. Apart from food products obtained from these sources, all other products are imported. The Faroe Islands do not have any natural trees or forests, which eliminates the possibility of wildlife (and their possible acquisition as food).
2. Meat obtained from pilot whales can be considered organic: animals live freely, feeding on natural food, without human intervention in their health (so they are not given drugs, probiotics, substances stimulating growth, etc.) and natural selection ensures the elimination of sick and weak individuals.
3. Meat is obtained in a humane manner. The legal regulations currently in force were introduced as a result of the intervention of pro-animal activists, and the regulations assume, among other things, that only trained people are entitled to kill pilot whales. Animals are killed only in a controlled way, i.e., by cutting the spinal cord and blood vessels, followed by cutting the vessels in the neck of the animal (10, 55).
4. Supporters of hunting argue that other factors resulting from anthropopressure cause much stronger interference in the population of pilot whales than the hunting itself (19). Hunting kills around 1,000 animals per year, while global warming, pollution of sea basins, fisheries-related maritime traffic, destruction of the habitat and food resources of pilot whales and other cetaceans cause damage to the entire population to an extent that cannot be determined and measured. Research has shown that cetaceans react to human interference in their environment by increasing cortisol levels in their organisms (49).
5. Products obtained from pilot whale hunting are free food that is distributed among grindadrápers. This is intended to serve as an integration activity, but above all to provide free food to people who need it. The fact is, however, that these raw materials are then resold by members of the community, so it is not true that the meat and fat of pilot whales are not of commercial value.
6. Pilot whale hunting is not considered cruel by its supporters, and who regard that this activity is presented in a bad light and that the reports are not based on facts but only on images and exaggerated interpretations.
7. Pilot whale hunting is a better form of meat production than industrial animal husbandry.
8. Hunting of pilot whales is just like hunting of other animals that takes place outside of the Faroe Islands.
9. Grindadráps can be viewed as anti-globalization, a type of economic activity that contests global production systems imposed by corporations (56).

From the point of view of the opponents, the following arguments are raised in favor of the necessity to revise the legitimacy of pilot whale hunting:

1. The traditional demand for nutrients found in pilot whale meat and fat resulted from food deficits and difficulties in the selection of appropriate food products. These phenomena were particularly important due to the geographical location of the Islands, which were isolated from the mainland, making the supply of food difficult for a long time. There are no agricultural crops (due to the poor rocky soil) on the Faroe Islands. In the absence of other food supplies, fish, marine mammals, and crustaceans were the main component of the diet of the traditional Faroese. This diet was a form of adaptation to the environmental requirements. Today, however, the Faroe Islands' supply of food products is excellent, making it possible to assume that the consumption of pilot whale meat and fat is only a marginal source of food.
2. Pilot whale meat and fat have constituted the Faroese's diet for centuries, being basically the only source of animal protein available. Faced with civilisational changes resulting in the pollution of sea basins, it appears, however, that scientists' recommendations concerning the consumption of these resources are currently clearly negative: pilot whale meat should not be consumed by humans because of the accumulation of harmful substances in their tissues. Levels of mercury have been studied in particular. Consumption of pilot whale meat by pregnant women has a significantly negative impact on the child's health. Research that has been conducted in this field since 1977 indicates that the meat, fat, and internal organs of pilot whales (kidneys and liver) are contaminated (54). Mercury present in pilot whale meat has a negative influence on the development of the central nervous system in children, and the influence of its presence is detected as early as during adolescence. Mercury consumed with mother's milk also causes an increase in blood pressure and impairs the immune system. The accumulation of harmful elements in pilot whale meat increases the risk of Parkinson's disease, hypertension, atherosclerosis as well as type 2 diabetes and insulin secretion disorders in the elderly. Elderly people who in their childhood and adolescence consumed large amounts of traditional dishes, including pilot whale meat, are particularly vulnerable to the above mentioned diseases (57). Pilot whale meat and fat accumulate significant amounts of mercury and cadmium during the life of an animal (57). The presence of mercury does not show immediate toxic effects for the animals themselves, but its amount increases with age (58). The level of these elements in the bodies of long-finned pilot whales is higher than in other marine mammals (59). In 2008 the Faroese Hospital System released a recommendation to eliminate whale meat and blubber from human consumption. Most likely it was this opinion which led to the grindadráp not being held that year, but only that year (5).
3. The meat is obtained in an inhumane way: the stress accompanying the killing of pilot whales, the long hours of chasing, the killing of the animals so quickly which lead to the impossibility of controlling the process, and the lack of veterinary supervision—all of these factors determine the unequivocally inhumane course of the process.
4. The pilot whale meat was traditionally distributed among the community, but today it is also available for purchase and can be treated as a tourist attraction. The importance of obtaining free meat is decreasing due to the high standard of living of both the Faroese and Danes in general.
5. The tradition of killing pilot whales does not comply with the level of civilisational development of the twenty-first century community. It is inappropriate for the contemporary way of perceiving relations between humans and non-humans and is based on cruelty, which has no particular and practical basis. The use of modern technologies and solutions (motorboats, special knives for the killing of animals and neoprene suits ensuring thermal comfort for hunters) raises the question of the “authenticity of the tradition.”
6. Lack of respect for the animals that are being slaughtered, reflected in the behavior of both adults and children (e.g., jumping on killed animals). As Bulbeck and Bowdler (6) demonstrate, the attitude of hunters toward these animals can hardly be described as relational (in terms of maintaining respect for non-human animals; it would be more appropriate to call this attitude exploitative, oriented toward the maximum harvest).
7. Contemporary knowledge indicates that we still do not have a full picture of who pilot whales are and who is killed in the hunting process. Admittedly, from the point of view of activists there is no significant level of evolutionary development of the animal, but the fact of fighting to preserve its life as an autotelic value. The awareness, however, that pilot whales are very highly evolved animals should stimulate reflection on whether it is really necessary to consume them.
8. Opponents of the grindadráp emphasize that today the key element of this tradition seems to be the process of rushing the animals into the bay and their slaughter. The rest of the process does not gather so many participants or observers. So the celebration is about the killing itself and not about rituals or other types of rites. It should be emphasized that even in the historically recognized tradition of these hunts, it was believed that women should not participate in the hunts and that pregnant women should not even look at the animals, because their presence was supposed to cause the whales to swim back to the sea (6). The integrative function of this tradition could therefore be carried out effectively only in relation to a part of the community.

Interpretations of Faroese attitudes during the grindadráp indicate that their behavior, especially that of young men, is interpreted as non-chalant toward the animals; it is disrespectful and directed at playing and participating in group activities directed against animals. These behaviors are characterized by laughter, loud screams, and playful character (throwing objects at each other and chases at the waterfront immediately after the hunt). These are unstructured, subjective interpretations, but they do indicate how the observer can interpret these behaviors. However, such an assessment can also be found in media reports, which may indicate that they are correct. Some anthropological observations confirm that pilot whale hunting is presented as heroic, nationalistic, and in a masculinist style. Perhaps this is associated with a kind of van Gennep's rite of passage, or perhaps it is the result of the psychological tension associated with killing. However, this kind of behavior is difficult to reconcile with the ideal of a Scandinavian hunter who kills animals to feed his family without ever treating it as a game. In this perspective, hunting is always hard work, based on the respect for an animal killed in a “one to one” situation, which also requires a significant contribution of one's own involvement and means that one never kills more than necessary (also for pragmatic reasons: the need to trim and transport the animal). Grindadráp is a paleolithic type of hunting when the herd is driven into the abyss, without control over the quantity and quality or the type of animals being killed. Control after death, in the form of measuring, weighing, and classifying into specific categories—is yet another procedure that creates the impression of ensuring the welfare of animals (42, 48). Fielding (5) obtained results showing that young people still value food products obtained through whaling and that the food products remain popular among the young. This result might suggest a future trend, that continuing the tradition is important as heritage to the young. Elsewhere Fielding (36) stated that continuance of the tradition may be threatened by the interaction of several factors such as over-extraction through poor management, international protests of environmental activists, and the marine pollutants found in the tissues of the whales. This last factor may significantly influence the willingness of the Faroese to consume pilot whale products (57). Consumption of products, however, is just one side of the issue. The other is the tradition of killing whales. These two processes can be easily separated, and a rational (rationalized) justification for the continuation of killing the whales can be easily constructed.

## Actions Aimed at Preventing Pilot Whale Hunting

Opponents of grindadráp engage in numerous protests aimed at blocking the hunt [Greenpeace's active efforts in the 1980's which were later abandoned due to pressure to grant permission to cultivate traditions; (6)]. The protests are direct actions aiming at deterring the inflowing pilot whales, which are dangerously approaching the shore or disturbing the hunt itself. These actions should be treated as a classic civil disobedience in the meaning of Henry David Thoreau, as hunting for pilot whales is legal under Danish law.

Very close monitoring and documentation are other measures taken to block the hunt. These are instruments of social control and also tools for exerting pressure on the public opinion. By disseminating knowledge and information about the course of the hunt, the number of animals killed, the process of their trimming and the accompanying events, the general public gains insight into the actual relationships between humans and non-humans in the Faroe Islands.

Attempts were also made to pay for the discontinuation of pilot whale hunting. In September 2018, the Sea Shepherd organization submitted a financial offer to the Faroese Parliament. In exchange for the discontinuation of pilot whale hunting, the parliament was to receive £910,000—every year for the next 10 years. The offer was rejected.

Publicizing information about the grindadráp in social media on one hand provides information to the public by presenting views of animal rights defenders and, above all, providing professional, objective information about the hunting. On the other hand most objective information may be obtained from scientific publications and research.

## Media Coverage on Grindadráp

The Faroese media present reports of grindadráp in two ways. First of all, in a veiled form: no views of blood and views of killed animals, especially pregnant mothers, are avoided; the focus is placed on showing photographs of young, engaged people (mainly men) cooperating in pulling the ropes, driving motorboats, etc. Photographs presenting the trimming of the carcasses or (especially) the disposal of the carcasses are not shown. The event is presented in terms of a social festival, uniting people in a joint action and integrating a multi-generational community, including children (5). The photographs also show a part of the community, apparently not participating but standing in the distance and observing the events (56). Such a strategy may result from the actual perception of the hunt, but it may be driven by a kind of caution dictated by the pressure of international public opinion and the desire to avoid controversial discussions.

The second way the Faroese media presents the discussion about grindadráp is to place the entire phenomenon in the context of a conspiracy theory, which suggests the presence of significant social forces (eco-terrorists) who try to make decisions about Faroese traditions and culture (Local issue no 3/2019).

The media interpretation outside the Faroe Islands is completely different, explicitly presenting the grindadráp in headlines as mass slaughter and the murder of animals. As Bulbeck and Bowdler (6) indicate, a semantic analysis of press releases on the hunt reveal significant differences in the choice of vocabulary used. While opponents of hunting use terms such as massacre, murdering, or butchers, the supporters of hunting employ words such as production, acquisition, population management, or harvest, etc.

## Conclusions

In the context of the information presented, especially the medical and veterinary information, the hunt of long-finned pilot whales fulfills the criteria of ritual slaughter for the following reasons: firstly, the fact that this method of killing animals is rooted in tradition and perceived as an important cultural element, and, secondly, the technique adopted is a binding technique, in which the animal remains conscious and dies without stunning as a result of exsanguination. According to the most contemporary scientific research, such a method of killing animals causes significant stress and pain. After breaking the spinal cord, the animal's motor functions are impaired, so it cannot escape or defend itself, but the animal still maintains an awareness of what happens and of the further actions to come. This method of killing animals is ethically and morally unacceptable, according to our knowledge of animals as sentient beings (60–64). Unfortunately, common knowledge about the actual act of killing is superficial. Outside observers may think that paralyzing an animal after a spinal cord rupture is synonymous with the animal's immediate death. It is not. Dying may last even longer than 4 min (47). What seems to be very controversial, then, is the fact of the inhumanely long process of the hunt—it takes several hours to the chase the animals to the location, in order to find the most suitable place for their slaughter.

In turn, the fact that the tradition itself is subject to transformation and modification (e.g., still in 1927 the chasing of the animals to the bays was performed by means of rowboats, while today motorboats are used). In the past, the grindadráp participants had to use their everyday clothes to insulate themselves from the low water temperature. Today wet suits are used for better insulation. In 2014, regulations were introduced on the permitted method of pilot whale slaughter, which explicitly and legally excluded the use of other methods. The North Atlantic Marine Mammal Commission Instruction Manual on Pilot Whaling indicates that it is possible to introduce changes and that it is up to the people to agree on how far they should go. The lack of an economic justification for the necessity of the pilot whale slaughter places this practice in the sphere of traditional symbolic activities, which are reproduced only on the basis of historically established conventions but which may be amended by 'social contract' or a socially generated redefinition of this custom. As pointed out earlier, the processual nature of the changes in tradition may also include intentional modifications. There are no obstacles to such a modification process in relation to the grindadráp.

Public opinion from outside the Faroe Islands seems to have a negative perception of pilot whale hunting, as reflected in the nature of media reports. The opinion in this respect is also not heterogeneous in the Faroe Islands.

It is apparent that pilot whale hunting should be compared with wildlife hunting organized in other parts of the world, and not with industrial farming, although the moment of killing is very similar in ritual slaughter as in the grindadráp.

Ethical opposition is also raised by the fact that the level of intellectual and social development of long-finned pilot whales and the examination of metabolic processes of their organisms indicate that hunting is a source of enormous long-term stress for pilot whales, which is something the whales themselves are well aware of.

It would be interesting to know what those who are participating in the grindadráps know about the long-finned pilot whales who are being killed: Do they know the facts about the specificity of the animals' functioning? Do they understand their ability to feel and understand the world? Do they know what the social and psychological functioning of these animals is all about? These are questions regarding the level of factual knowledge and not about the level of empathy which cannot be significant if people kill animals with their own hands. It seems, however, that in such situations, an effective method of modifying attitudes is to start the process from providing objective information that broadens the spectrum of knowledge of those involved in the grindadráp.

It would also be interesting to know to what extent those who have direct contact with and participate in the grindadráp perceive their participation as an act of killing. The Instruction Manual on Pilot Whaling, which came into force in 2014 (after the Sea Shepherd Operation Grindadráp Stop), ordered that beginning in 2015, whalers who participate in the killing process—as distinct from other aspects of whaling— have to be certified as having attended a training course on the subject (10). This kind of regulation automatically sets rules about who can actively participate in the grindadráp and at which stage they can join the process. The roles, the flow, and the actual rules regulating participation in the grindadráp are described in detail (10, 39). The authors describe all practical regulations and rules of the grindadráp in a very detailed way: “The first grindadráp aformann (the singular form of the word) to reach the pod of whales by boat hoists the white, red, and blue Merkið* or Faroese flag, on his boat's mast, thus signaling his leadership role in the ensuing grindadráp” [(10), p. 42].

Legal regulations (North Atlantic Marine Mammal Commission Instruction Manual on Pilot Whaling) indicate clearly who can cut the spinal cord and blood vessels. However, to what extent are people pulling the ropes hooked in pilot whales' blowholes aware of the act of killing an animal? Do they think that they are actually killing the animal or that they are helping to pull the animal ashore? Theoretically, one can recreate here a kind of psychological process of distancing oneself from killing in favor of emphasizing participation in community activities (5). The stages of pilot whale hunting can be distinguished as: observing the sea; identifying the presence of the pod; preparing equipment; sailing out on boats; driving the pod into bays; hooking; pulling animals ashore; immobilizing; inserting a lance that cuts the spinal cord; cutting through body shells and blood vessels; transporting the dead animals to the harbor; trimming the carcasses, including measuring, describing, and taking samples for research purposes and the trimming of the meat and fat and disposing of leftovers. It turns out that very few people actually participate in the act of killing. All others can say, according to how they want to interpret the situation, is that they are either killing the animals or not killing them.

The perception of human relationship to the world of non-human animals, including humans and pilot whales, depends directly on the level of consciousness and empathy of humans. Raising the level of these psychological competencies seems to be the only way to improve the welfare of non-human animals and perhaps to have an effective impact on the introduction of a prohibition on cetacean hunting (42). The whales, on the other hand, as socially, psychologically and somatically highly developed organisms, are aware of the human activities to which they are exposed. They experience them at the somatic and psychological levels and actively try to defend themselves against these actions. In the light of veterinary, zoological, and psychological knowledge, pilot whale hunting should be treated unequivocally as an act of abuse on non-human animals.

This perspective may be seen as contradictory to the perception of pilot whale hunting in the Faroe Islands, where, however, opinion is not consistent. To the contrary, it is polarized (65). Not all of the residents are in favor of the grindadráp. Fielding (5) predicts that hunting pilot whales will remain popular as food products obtained through whaling remain popular among the educated youth in the Faroese Islands (more young males than females consume whale products). Seventy-eight percent of respondents in his research believed that whaling will continue in the Faroe Islands. The author also found that a majority of students make an effort to attend the grindadráp if possible. Moreover, half of the male respondents actively participate in the hunt. In his survey Fielding asked his subjects an open question about the future of whaling in the Faroe Islands. He obtained a “more nuanced response in the Faroe Islands, with half of the students saying that it will and one-third ‘hopeful' but not fully confident” Fielding [(5), p. 13]. Research demonstrates that certain types of social events (like the Faroese Hospital System statement about the healthiness of whale products or the 2014 Sea Sheperd Operation Grindadráp Stop) influence social practices and consumer choices. Therefore, opinions of the Faroese on whaling as a type of activity important for local culture also change. Practically no young persons in the Faroe Island can portray themselves as professional whalers [only 1% of students indicated that they would consider such a career, while more than 97% answered negatively, (5)].

Reflecting before on setting the borderline between acceptable and non-acceptable traditions I proposed “setting a limit based on whether the practices in question involve physical damage to the shells of a human body or another animal against their will.” Following this definition grindadráp cannot be considered an acceptable cultural practice as it causes obvious damage to the physical integrity of an animal's body, which also means violation of an animal's will to live.

## Author's Note

This article in initial form was also published as “Ubój rytualny—tradycja polowania na grindwale na Wyspach Owczych.” In H.Mamzer (ed.) (2020). Róznice kulturowe w traktowaniu zwierząt. Atut. Wrocław, pp. 95–129. Reprint of this Polish version was published as: “Ubój rytualny—tradycja polowania na grindwale na Wyspach Owczych,” part. 1 in Życie Weterynaryjne (2019) no 94(11), pp. 753–758 and “Ubój rytualny—tradycja polowania na grindwale na Wyspach Owczych” part. 2 in Życie Weterynaryjne (2019) 94(12), pp. 814–820.

## Author Contributions

The author confirms being the sole contributor of this work and has approved it for publication.

## Conflict of Interest

The author declares that the research was conducted in the absence of any commercial or financial relationships that could be construed as a potential conflict of interest.

## Footnotes

^1^Longitudinal studies indicate hormonal responses to stress caused by chronic anthropogenic factors such as climate change, freshwater discharges to seas, pollution, and many others [see Trumble et al. (49) and de Vere et al. (19)].
